# Facile synthesis of composition-tuned ZnO/Zn_*x*_Cd_1-*x*_Se nanowires for photovoltaic applications

**DOI:** 10.1186/s11671-015-0886-3

**Published:** 2015-04-15

**Authors:** Qiang Luo, Zhiming Wu, Jialun He, Yiyan Cao, Waseem Ahmed Bhutto, Weiping Wang, Xuanli Zheng, Shuping Li, Shengquan Lin, Lijing Kong, Junyong Kang

**Affiliations:** Department of Physics, Fujian Key Laboratory of Semiconductor Materials and Applications, Xiamen University, 422 Siming South Road, Xiamen, 361005 People’s Republic of China; Department of Automation, Xiamen University, 422 Siming South Road, Xiamen, 361005 People’s Republic of China

**Keywords:** ZnO/ZnCdSe coaxial nanowires, Composition tuning, Alternant physical deposition, Solar cell

## Abstract

ZnO/Zn_*x*_Cd_1-*x*_Se coaxial nanowires (NWs) have been successfully synthesized by combining chemical vapor deposition with a facile alternant physical deposition method. The shell composition *x* can be precisely tuned in the whole region (0 ≤ *x* ≤ 1) by adjusting growth time ratio of ZnSe to CdSe. As a result, the effective bandgaps of coaxial nanowires were conveniently modified from 1.85 eV to 2.58 eV, almost covering the entire visible spectrum. It was also found that annealing treatment was in favor of forming the mixed crystal and improving crystal quality. An optimal temperature of 350°C was obtained according to our experimental results. Additionally, time resolved photo-luminescence spectra revealed the longest carrier lifetime in ZnO/CdSe coaxial nanowires. As a result, the ZnO/CdSe nanowire cell acquired the maximal conversion efficiency of 2.01%. This work shall pave a way towards facile synthesis of ternary alloys for photovoltaic applications.

## Background

One-dimensional nanostructures have attracted considerable attention due to their unique advantages and potential applications in photovoltaic devices [1-3]. In particular, nanostructured oxide semiconductors, such as ZnO nanowires (NWs) and TiO_2_ nanocrystals, have been widely applied to photo-electrochemical (PEC) cells or solar cells owing to the low cost and high stability against photocorrosion, and mature fabrication techniques [4-13]. However, these oxide semiconductors have a relatively wide bandgap and can not efficiently absorb sunlight in visible region, yielding a low efficiency. A series of semiconductor nanocrystals, such as ZnSe [14], CdSe [15], CdS [16], CdSeTe [17], ZnCdSe [18], and ZnCdTe [19], have been coated onto the surface of ZnO or TiO_2_ to expand photoresponse. As compared with binary alloys, ternary materials are the more efficient sensitizers due to their tunable bandgaps and band structures [17-26]. Many efforts have been devoted to tune their compositions by different fabrication methods. For instance, Xu et al*.* fabricated type II ZnO/Zn_*x*_Cd_1-*x*_Se nanocables via an ion-exchange approach [27]; Ruchi et al*.* prepared TiO_2_/Zn_*x*_Cd_1-*x*_Se nanotubes through a successive ionic layer adsorption and reaction technique [28]; Li et al. synthesized Zn_*x*_Cd_1-*x*_Se shell layer on ZnO NWs by chemical vapor deposition (CVD) method [25]. In spite of these efforts, the composition of ternary alloys can not be conveniently controlled because of the different ion concentrations in solution method or the different saturated vapor pressures of elements in vapor method. Hence, up to date, it is still a challenge to develop a simple and facile route to fabricate composition-tuned ternary alloys.

In this work, we successfully synthesized Zn_*x*_Cd_1*-x*_Se shell layers on ZnO NWs with tunable compositions (0 ≤ *x* ≤ 1) by an alternant physical deposition method. The scanning electron microscopy (SEM), high-resolution transmission electron microscopy (HRTEM), X-ray diffraction (XRD), transmission analysis and time resolved photo-luminescence (TRPL) were performed to investigate their morphologies, crystal structures, compositions, and optical properties, respectively. It has been found that the composition of ZnCdSe shell could be conveniently and precisely controlled by adjusting growth time ratio of ZnSe to CdSe. Meanwhile, solar cells based on different ZnO/ZnCdSe coaxial NWs were assembled and their performances were evaluated as well. This work opens a novel avenue for facile synthesis of sophisticated ternary alloys.

## Methods

### Synthesis of ZnO NWs and Zn_*x*_Cd_1-*x*_Se shells

**Figure 1:**
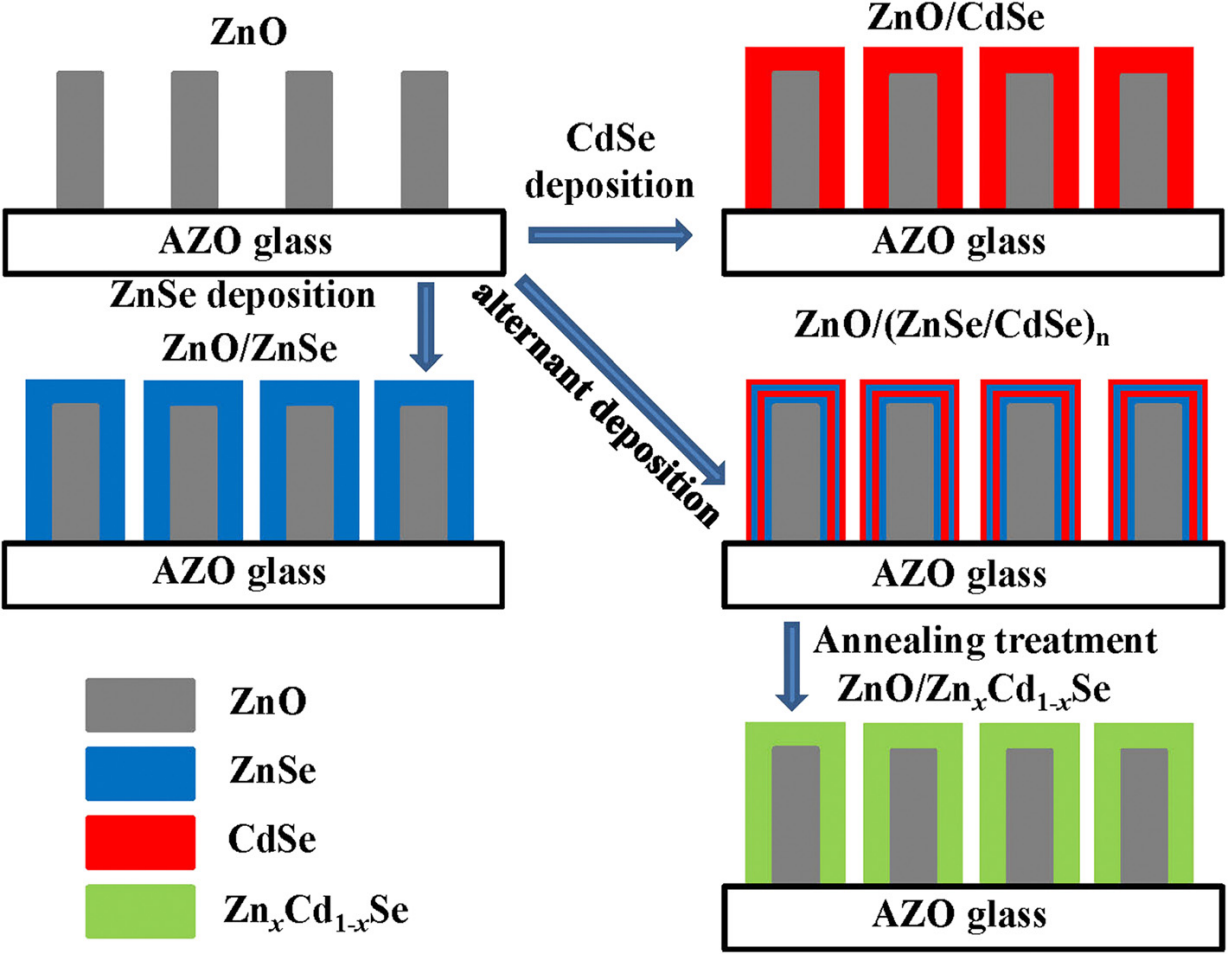
Schematic illustration for the fabrication of ZnO/Zn_*x*_Cd_1-*x*_Se coaxial NWs.

ZnO NWs were grown through CVD method, which has been reported in our previous work [14]. The Zn_*x*_Cd_1-*x*_Se shells were deposited on ZnO NWs by alternant radio-frequency (RF) magnetron sputtering of the ZnSe (99.99%) and CdSe (99.99%) targets in an Ar ambient. Prior to the deposition, the vacuum chamber was evacuated down to 3 × 10^−4^ Pa. During the growth, the working pressure was maintained at 0.8 Pa and the sputtering power was set at 80 W. The growth rates of these two materials were identified to be about 0.16 nm per second. Strategy for the synthesis of Zn_*x*_Cd_1-*x*_Se shells was illustrated in Figure 1. To fabricate ZnCdSe shell, multi-layers of ZnSe/CdSe were first grown by alternant sputtering deposition and then were annealed to form ternary alloys. Different compositions can be facilely achieved by controlling the growth time ratio of ZnSe to CdSe. In our experiment, six different cases were performed with the growth time (*t*_1_:*t*_2_) of 0 s:30 s, 6 s:24 s, 12 s:18 s, 18 s:12 s, 24 s:6 s, 30 s:0 s, where *t*_1_ and *t*_2_ represent the growth time of ZnSe and CdSe in every cycle, respectively. These samples were labeled as I, II, III, IV, V, and VI, respectively. The whole growth procedure lasted for 10 cycles, and the as-grown samples were annealed under 350°C and 400°C

### Device assembly

For photovoltaic applications, the as-prepared ZnO/Zn_*x*_Cd_1-*x*_Se coaxial NWs were used as the working electrodes. Nanostructured counter electrode was prepared by sputtering a thin layer of Cu_2_S on aluminum zinc oxide (AZO) glass. The two electrodes were sealed together with a 60-μm-thick polypropylene spacer (Surlyn, DuPont, Wilmington, USA), and the internal space of the cell was filled with a polysulfide electrolyte (1.0 M S, 1.0 M Na_2_S, and 0.1 M NaOH in deionized water). The active area of the solar cell was about 0.5 cm^2^.

### Physical characterization

The morphologies of the as-prepared ZnO and ZnO/Zn_*x*_Cd_1-*x*_Se NWs were measured with a field emission SEM (LEO 1530, Zeiss, Thornwood, USA). The structures and compositions were characterized by XRD (X’Pert PRO, PANalytical, Chapel Hill, USA) and TEM (Tecnai F30, Fei, Hillsboro, USA). The transmission spectra were measured using a Varian Cary 5000 UV-vis NIR spectrophotometer (Agilent, Santa Clara, USA). TRPL measurements were carried out in an Edinburgh FLS920 spectrofluorometer (Edinburgh Instruments Ltd, Livingston, UK) at room temperature. The detected energies for different samples were in accordance with their estimated bandgaps (1.85, 1.98, 2.05, 2.18, 2.34, and 2.58 eV). Current density-voltage (*J*-*V*) characteristics of solar cells were recorded under AM1.5 solar illumination (100 mW **·** cm^−2^). The incident photon-to-current conversion efficiency (IPCE) was measured on a broadband spectroscopy system consisting of a grating monochromator (Spectra Pro-750i, Acton Research Corporation, Trenton, USA), a 100 W bromine-tungsten lamp, and a lock-in amplifier (SR830 DSP, Stanford Research Systems, Sunnyvale, USA), by comparing with a reference Si and Ge cells.

## Results and discussion

**Figure 2:**
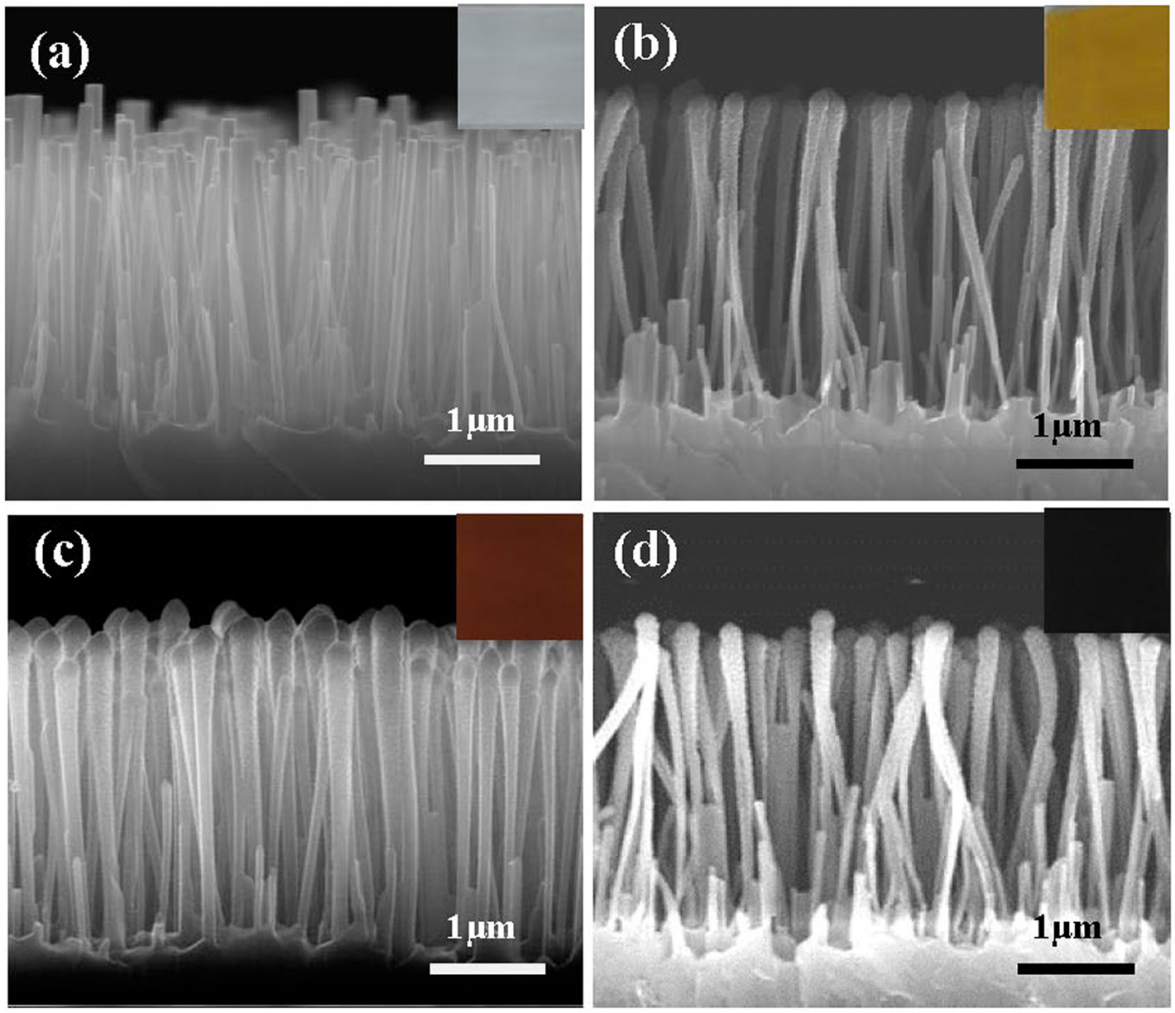
Cross-sectional SEM images. **(a)** Bare ZnO NWs; **(b)** ZnO/ZnSe coaxial NWs; **(c)** Zn*x*Cd1-*x*Se coaxial NWs; **(d)** ZnO/CdSe coaxial NWs. The insets in **(a-d)** are their photographs.

The morphologies of bare ZnO NWs and ZnO/Zn_*x*_Cd_1-*x*_Se coaxial NWs were investigated by SEM. Figure 2a shows a typical SEM image with a cross-sectional view of ZnO NWs, exhibiting the vertical growth and smooth surface. After the deposition of Zn_*x*_Cd_1-*x*_Se shells, the nanowire surfaces become rough as presented in Figure 2b,c,d. Generally, the coaxial NWs fabricated by physical method show a pyramidal shape due to the easier deposition at the top position [29]. At present work, the relatively uniform shell layers were attained by optimizing the working pressure and power, which was beneficial for light absorption and carrier separation. The insets in Figure 2 show their photographs. One can see that the sample color gradually changes from yellow (ZnSe) to red (Zn_*x*_Cd_1-*x*_Se) and then to dark (CdSe), revealing the graded composition in shells.

**Figure 3:**
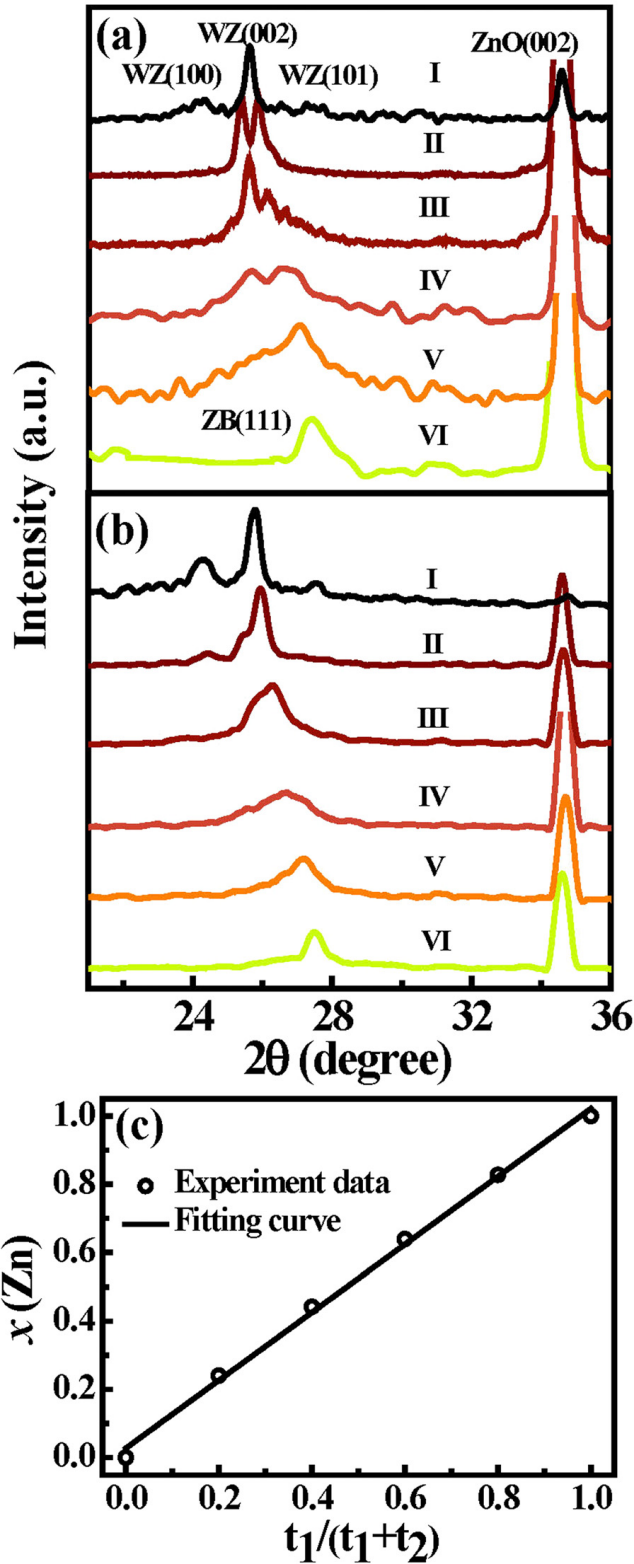
XRD patterns and dependence of the Zn content on growth time ratio. **(a)** XRD patterns of as-grown ZnO/Zn*x*Cd1-*x*Se coaxial NWs; **(b)** XRD patterns of ZnO/Zn*x*Cd1-*x*Se coaxial NWs annealed under 350°C; **(c)** the dependence of the Zn content in the ZnCdSe shells on the growth time ratio.

To elucidate the crystal structures and compositions of different samples, XRD measurements were performed. Figure 3a shows the XRD patterns for as-grown samples. For all the patterns, there are sharp peaks at around 34.6°, indexed to (002) plane of wurtzite (WZ) ZnO. After the deposition of shell layers, different peaks appear in the region from 24° to 28°. As for ZnO/ZnSe NWs (sample VI), the peak is located at 27.47°, corresponding to (111) plane of zinc blende (ZB) ZnSe (JCPDS 80–0021). The positions of diffraction peaks for ZnCdSe show a whole shift to the smaller angles with the decrease of Zn content. Meanwhile, additional peaks gradually appear, and three peaks at 23.92°, 25.42°, and 27.14° became clearly visible for ZnO/CdSe NWs (sample I), which are assigned to (100), (002), and (101) planes of WZ CdSe (JCPDS 77–2307), respectively. The evolution behavior originated from different stable phase structures of ZB and WZ for ZnSe and CdSe, respectively. As a result, the phase structure of ZnCdSe alloy will gradually change from ZB to WZ with the decrease of Zn content. It can bee seen that the composition *x* corresponding to phase transition is about 0.4, which is similar to other reports [30,31]. In addition, the XRD peaks of ternary ZnCdSe alloys are broader than those of binary alloy ZnSe or CdSe; moreover, there appear clearly double peaks in sample II. This phenomenon is attributed to the half-baked alloy behavior resulting from the alternant physical deposition under low temperature. In order to thoroughly alloy the multi-layers, we carried out the annealing treatment for the samples under different temperatures. Figure 3b shows the XRD patterns of samples annealed at 350°C. As compared with those in Figure 3a, the XRD peaks of ZnCdSe alloys became sharper, demonstrating the improved crystal quality. It is worth noting that these XRD patterns almost evolve from double peaks into single peak after annealing treatment, revealing the further alloying process for ZnCdSe shells. Based on the position and intensity of the diffraction peaks, the compositions *x* of Zn_*x*_Cd_1-*x*_Se alloys were estimated to be 0.24, 0.44, 0.64, and 0.83 for samples II, III, IV, and V, respectively [18,32,33]. Figure 3c shows the dependence of the composition *x* on the growth time ratio. The linear relationship suggests that the composition of ternary Zn_*x*_Cd_1-*x*_Se alloys has been precisely controlled.

**Figure 4:**
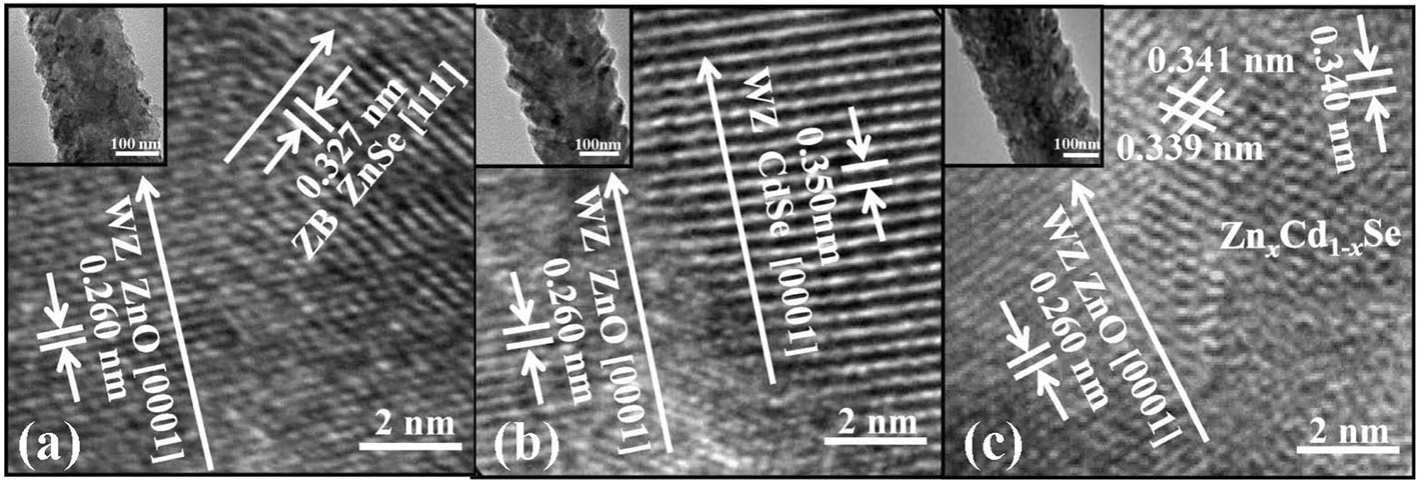
HRTEM images of **(a)** ZnO/ZnSe coaxial NW; **(b)** ZnO/CdSe coaxial NW; **(c)** Zn_0.24_Cd_0.76_Se coaxial NW. The insets in **(a-c)** show their low-resolution TEM images.

The structures of ZnO/ZnCdSe coaxial NWs annealed under 350°C were further investigated by TEM measurements. Inset in Figure 4a shows the typical low-magnification TEM image of a ZnO/ZnSe nanowire. The surface is fully covered by a layer of nanocrystals, confirming the successful fabrication of a coaxial structure. The HRTEM image in Figure 4a reveals the detailed structure of ZnO/ZnSe coaxial nanowire. The interplanar spacing of 0.260 nm in the inner core is identified to WZ ZnO with [0001] growth direction, while the fringe spacing of 0.327 nm in the outer shell is assigned to ZB ZnSe with [111] growth direction. An obvious tilt is observed between both growth directions owing to their large lattice mismatch, which is similar to other reports [34]. Figure 4b shows the HRTEM image of a ZnO/CdSe NW with an inset of low magnified image. The fringe spacing of 0.350 nm in the outer layer matches well to bulk WZ CdSe in *c*-axis direction. Due to the same crystal structures of ZnO and CdSe, there only exhibits a slight tilt between both [0001] directions; however, the large mismatch stress is mostly accommodated by the interface, resulting in the interfacial defects. As for the ZnO/Zn_*x*_Cd_1-*x*_Se NWs, we choose sample II (ZnO/Zn_0.24_Cd_0.76_Se) as an example to get an insight into the details. As shown in Figure 3c, the shell structure is quite different from that in Figure 3a,b. The shell layer is of polycrystalline in structure because of strained inducement [18]. The grains show different interplanar spacings and growth directions, which agree well with the discussion in XRD results. Notably, the fringe spacings of grains are larger than those of ZnSe, but smaller than those of CdSe, demonstrating the successful alloying by annealing treatment. It should be mentioned that the ZnCdSe alloy is obtained by alternant physical deposition and annealing treatment. Hence, the element distribution was seriously affected by diffusion process during heat treatment. In this sense, annealing temperature plays a key role on element distribution.

**Figure 5:**
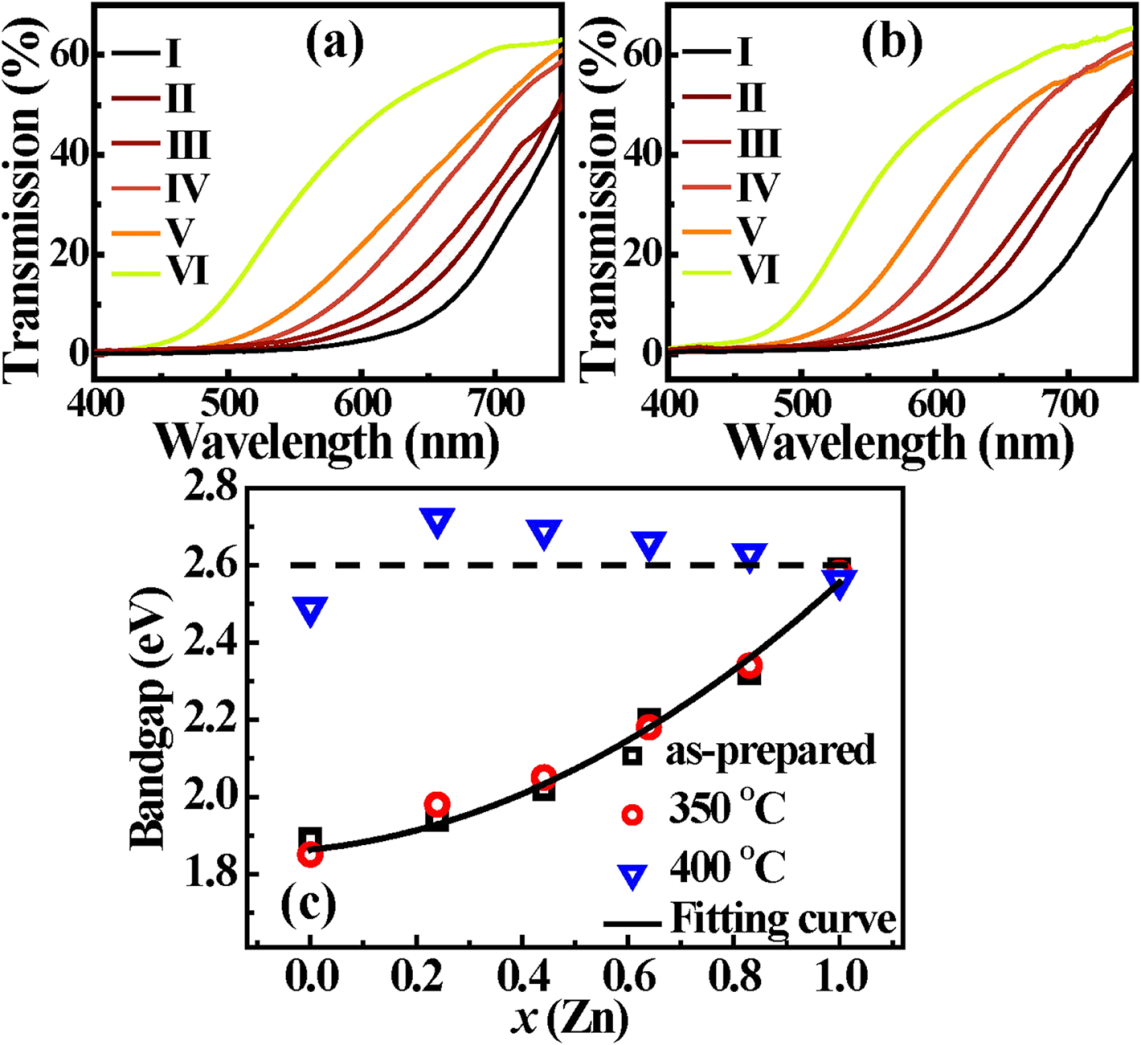
Transmission spectra and dependence of the bandgap on Zn content. **(a)** Transmission spectra of as-grown ZnO/Zn*x*Cd1-*x*Se coaxial NWs; **(b)** transmission spectra of ZnO/Zn*x*Cd1-*x*Se coaxial NWs annealed under 350°C; **(c)** the dependence of the bandgap on the Zn content of ZnCdSe shell.

To determine the optical characteristics of the ZnO/Zn_*x*_Cd_1-*x*_Se coaxial NWs, transmission measurements were performed. The bandgap was estimated by the onset of transmission curve. As for unannealed samples, it can be seen from Figure 5a that the bandgaps of Zn_*x*_Cd_1-*x*_Se alloys demonstrate a systematic blue shift from 1.85 eV (670 nm) to 2.58 eV (480 nm) with the increase of Zn content, almost covering the entire visible spectrum. After annealing treatment under 350°C, as shown in Figure 5b, the absorption edge of samples becomes steeper compared with that of unannealed ones, illustrating the improved crystal quality of ZnCdSe shells, which is consistent with the XRD results. To further understand the alloying kinetics, we plotted the dependence of ZnCdSe bandgaps on composition *x* for samples annealed under different temperatures. As shown in Figure 5c, the bandgaps of samples annealed under 350°C look the same as that of as-prepared samples, demonstrating that the alloy composition remains unchanged. As reported by others [18,27], the bandgap shows a nonlinear dependence on composition *x*, which was fitted by *Eg(x) = Eg(CdSe) + (Eg(ZnSe)–Eg(CdSe)–b)x + bx*^*2*^, where *b* is the bowing parameter. The least square fit yields *b* = 0.55 eV, which is slightly larger than that of bulk (0.41 ~ 0.48 eV), but lower than that obtained by Yoon (0.79 eV) [18,31,35]. As we know, bowing parameter *b* in a mixed crystal of A_*x*_B_1-*x*_C reflects their miscibility between AC and BC [30,36,37]. From this point of view, the lower *b* value in our work demonstrates that the ternary alloys annealed under 350°C have relatively uniform element distribution. Note that the bandgaps of alloys tend to keep at 2.6 eV when the annealing temperature increases up to 400°C, which is almost equal to that of bulk ZnSe. This behavior reveals that the alloy composition varies owing to the evaporation of Cd element under the higher annealing temperature. Hence, it is important to control the alloying process for the fabrication of ternary alloys.

**Figure 6:**
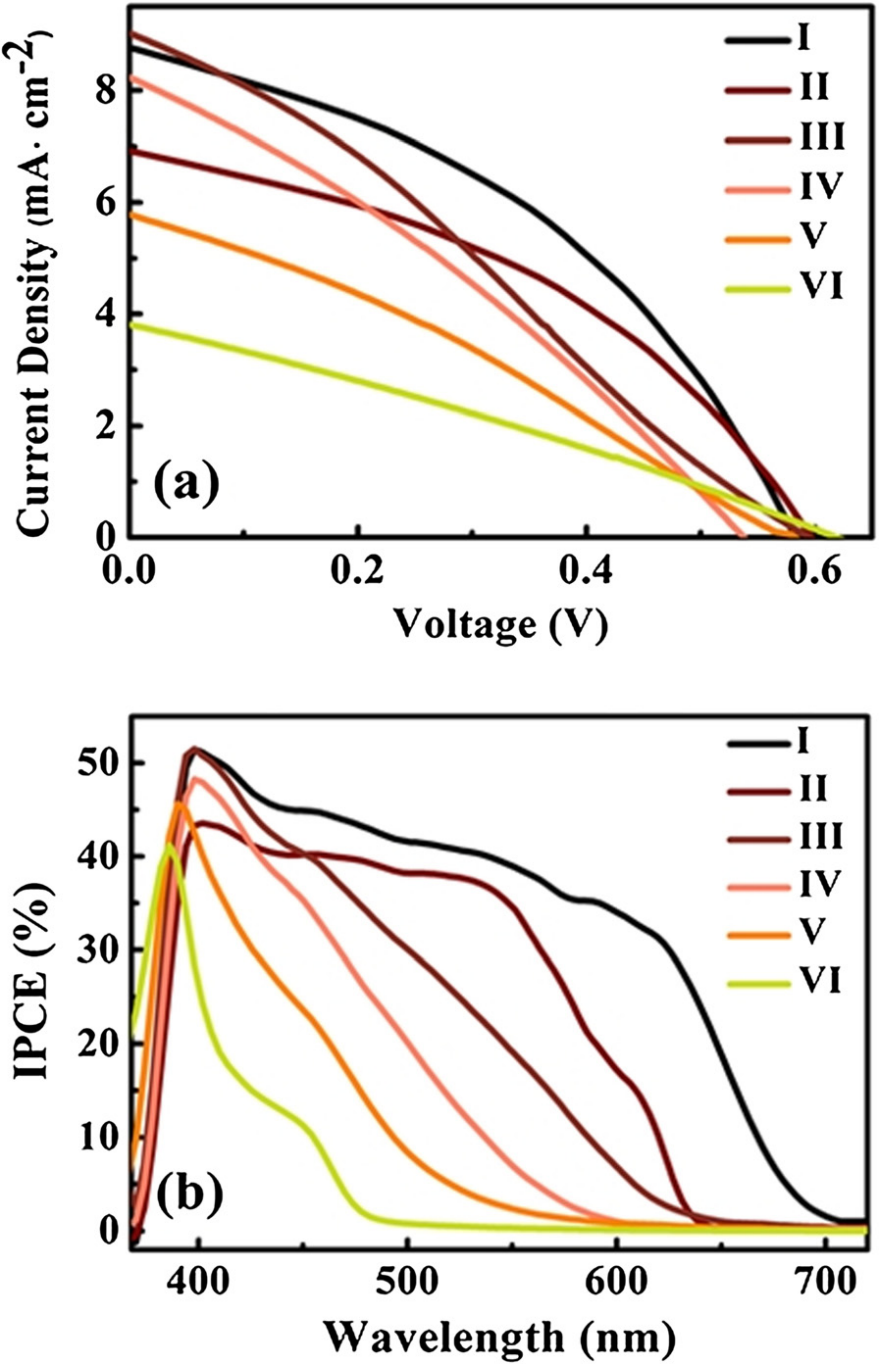
Current density-voltage characteristics **(a)** and IPCE curve **(b)** of PEC cells.

The PEC cells were fabricated by using the different samples. Figure 6a displays the *J*-*V* characteristic curves of the cells. The detailed performance parameters are summarized in Table 1. The ZnO/ZnSe nanowire cell provided the lowest conversion efficiency of 0.68%. With the decrease of Zn content, the conversion efficiency gradually increased and the maximal conversion efficiency of 2.01% was obtained in the ZnO/CdSe nanowire cell, corresponding to an open-circuit voltage (*Voc*) of 0.58 eV and *J*_*sc*_ of 8.75 mA · cm^−2^. Figure 6b shows the IPCE of the cells. The photoresponse threshold shifts gradually to the long wavelength with the decrease of Zn content, which is strongly linked with that of the transmission spectra.

**Table 1:** **Performances of solar cells measured under AM1.5 (100 mW · cm** ^**−2**^ **)**

**Number**	***x***	**Composition**	***J***_***sc***_ **(mA · cm** ^**−2**^ **)**	***V***_***oc***_ **(V)**	**FF**	***Ƞ*** **(%)**
I	0.00	ZnO/CdSe	8.75	0.58	0.39	2.01
II	0.24	ZnO/Zn_0.24_Cd_0.76_Se	6.90	0.59	0.39	1.63
III	0.44	ZnO/Zn_0.44_Cd_0.56_Se	9.00	0.59	0.28	1.53
IV	0.64	ZnO/Zn_0.64_Cd_0.36_Se	8.25	0.54	0.30	1.33
V	0.83	ZnO/Zn_0.83_Cd_0.17_Se	5.75	0.58	0.30	1.02
VI	1.00	ZnO/ZnSe	3.75	0.62	0.29	0.68

FF, fill factor.

**Figure 7:**
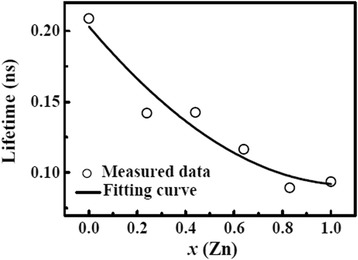
PL decay times for different samples annealed under 350°C.

It is known that conversion efficiency of type II solar cells significantly are related to multiple factors, including light absorption, charge separation, carrier recombination, etc. In principle, a narrow bandgap is beneficial for the light absorption and improvement of *J*_*sc*_; however, it is unfavorable for the charge separation due to the low band offset [38], resulting in the decrease of *Voc*. Hence, the optimal performance is generally achieved in the ZnO/Zn_*x*_Cd_1-*x*_Se cells with nonzero *x*. For example, Yoon et al. fabricated composition-tuned ZnO/Zn_*x*_Cd_1-*x*_Se nanowire cells and obtained a maximal solar efficiency of 1.5% at *x* = 0.3 [18]; whereas, Lin et al. reported Zn_*x*_Cd_1-*x*_Se quantum-dot-sensitized TiO_2_ solar cells, which showed the best performance at *x* = 0.6 [39]. To explain the best performance for the ZnO/CdSe nanowire cell in our experiment, TRPL measurements were performed to further gain the dynamic information on carrier separation and recombination. The PL decay curves were fitted by a typical biexponential form to obtain the average lifetimes τ [38]. As shown in Figure 7, the carrier lifetime of ZnO/Zn_*x*_Cd_1-*x*_Se coaxial NWs exponentially decreases with the increase of Zn content. The ZnO/CdSe NWs possess the longest lifetime, demonstrating the lowest recombination rate. Assuming the same diffusion coefficients (*D*) in the ZnCdSe alloys, the longer lifetime refers the longer diffusion length *L* ($L=\sqrt{D\cdot \tau }$) and thereby the higher carrier separation and collection efficiency. Based on the above analysis, the strong light absorption (due to the narrow bandgap) and the long carrier lifetime in ZnO/CdSe NWs dominantly contributed to the high conversion efficiency. Finally, it should be pointed out that the performance of nanowire cells also relies on the shell thickness. The increased shell thickness is helpful for the light absorption, but it will decrease the charge separation efficiency due to the increased diffusion length of the photon-generated carriers. In this work, the shell of 50 nm in thickness was grown according to the relevant reports [20,40], and further optimization is potential to improve cell performance. In the light of these, multiple factors, including effective bandgap, crystal quality, band structure, and shell thickness, etc., should be comprehensively considered to achieve a high-efficiency nanowire solar cell.

## Conclusions

In summary, we demonstrated that the ZnO/Zn_*x*_Cd_1-*x*_Se coaxial NWs with tunable shell compositions could be facilely synthesized by combining CVD with an alternant physical deposition method. Morphological studies by SEM show that the entire ZnO nanowire can be coated with a relatively uniform shell. XRD and TEM results disclosed that the composition *x* can be precisely controlled by adjusting growth time ratio of ZnSe to CdSe; meanwhile, annealing treatment under a suitable temperature is beneficial for forming ternary alloys and improving crystal quality. Transmission analysis indicated that the effective bandgap of ternary alloys could be modified in a wide range from 1.85 eV to 2.58 eV by composition tuning. Time resolved photo-luminescence spectra revealed that carrier lifetime of ZnO/Zn_*x*_Cd_1-*x*_Se coaxial NWs exponentially decreases with the increase of Zn content. A maximal conversion efficiency of 2.01% was achieved in ZnO/CdSe nanowire cell. This work provides a facile method to synthesis ternary alloys with tunable composition.
